# Supplementary material for: Mir526b and Mir655 Promote Tumour Associated Angiogenesis and Lymphangiogenesis in Breast Cancer
Source: Cancers (Basel). 2019 Jul 4;11(7):938. doi: 10.3390/cancers11070938 (PMC6678879; doi:10.3390/cancers11070938)
Supplement: Supplementary file 1 [file cancers-11-00938-s001.pdf]

# Supplementary Materials: Mir526b and Mir655 Promote Tumour Associated Angiogenesis and Lymphangiogenesis in Breast Cancer

**Stephanie Hunter, Braydon Nault, Kingsley Chukwunonso Ugwuagbo, Sujit Maiti, and Mousumi Majumder**

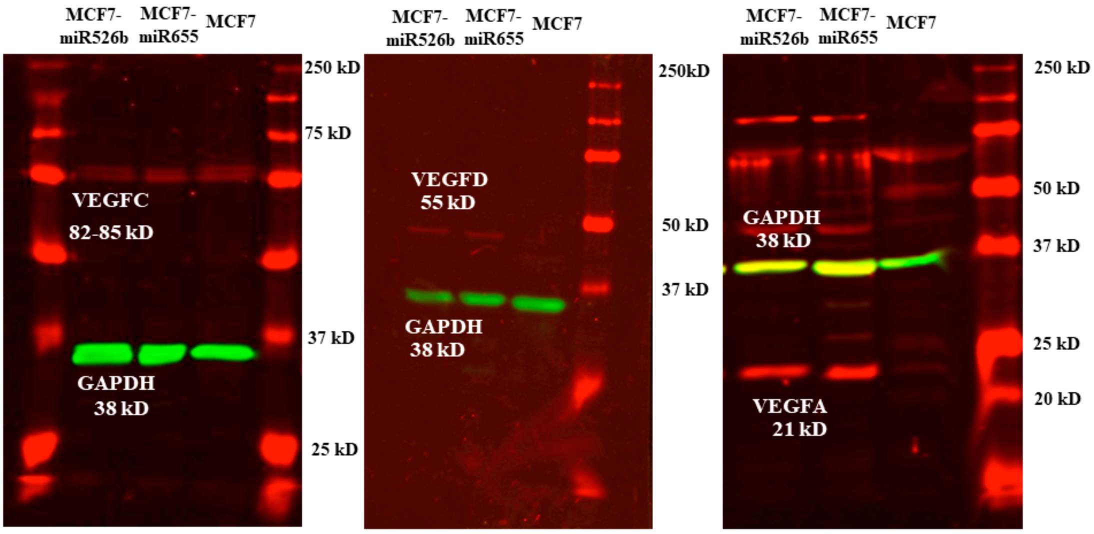


**Figure S1.** Western blot analysis of VEGFs in cancer cell lines. Overexpression of miR526b and miR655 results in an upregulation of angiogenesis and lymphangiogenesis markers VEGFA, VEGFC, and VEGFD at the protein level.

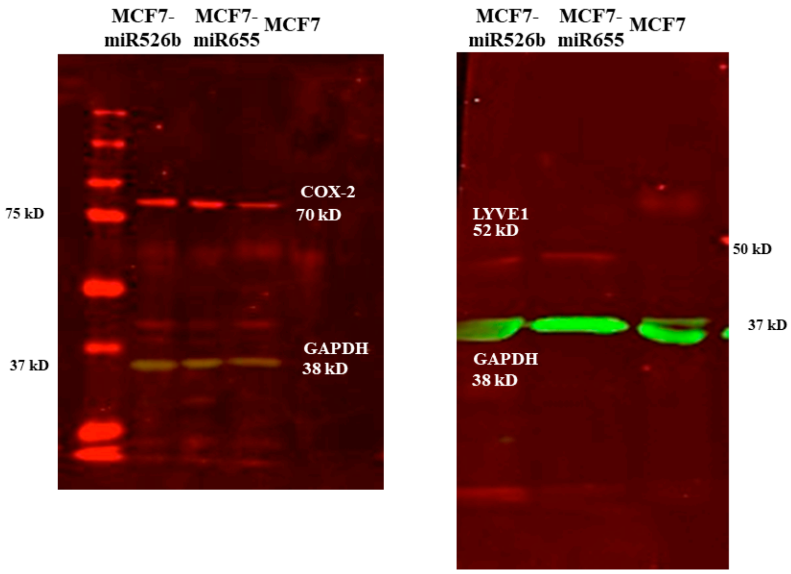


**Figure S2.** Western blot analysis of COX-2 and LYVE1 in cancer cell lines. Overexpression of miR526b and miR655 results in upregulation of COX-2 and lymphangiogenesis marker LYVE-1 at the protein level.

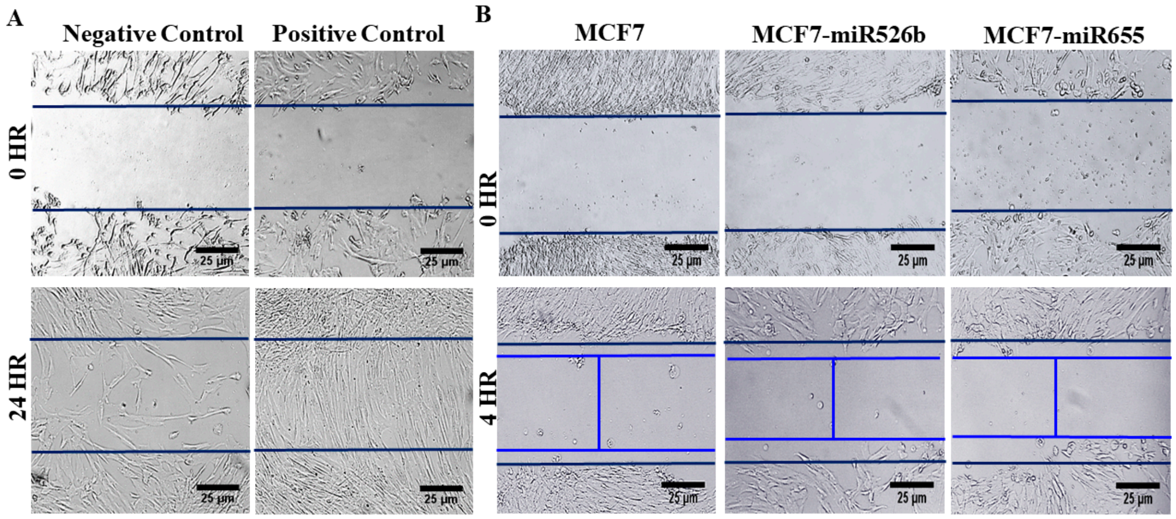


**Figure S3.** MCF7-miR526b and MCF7-miR655 conditioned media promotes cellular migration of HUVECs. (**A**) HUVECs treated with unsupplemented media serve as negative control. HUVECs treated with supplemented media serve as positive control. (**B**) HUVECs treated with miRNA conditioned media at other time points not included in Figure 3. Navy lines represent scratch wound boundary at 0 h. Light blue lines represent the migration front at given times.

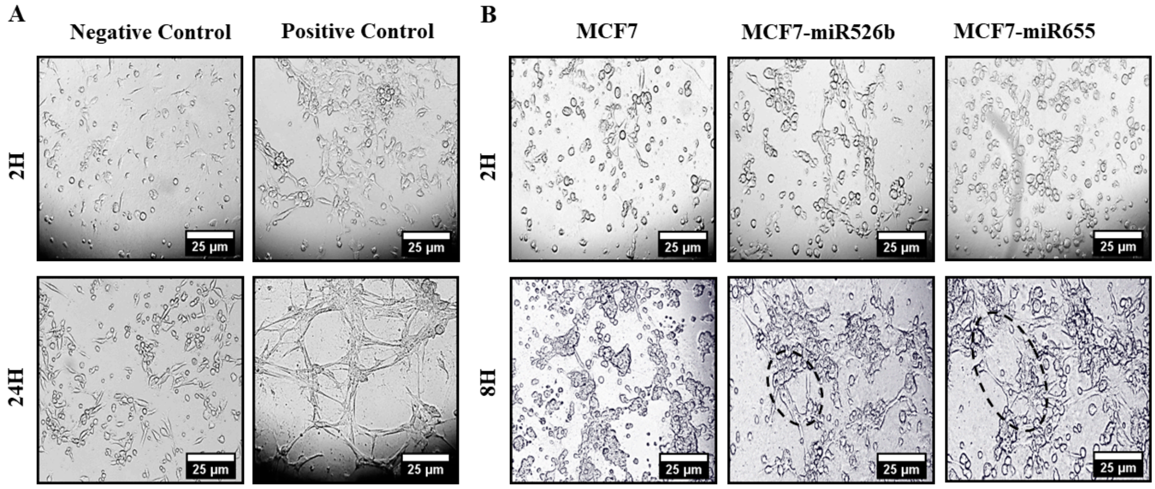


**Figure S4.** Secretions collected from miR526b and miR655 overexpressing cells results in an increase of tube formation of HUVECs. (**A**) HUVECs in unsupplemented media serve as negative control. HUVECs in supplemented media serve as positive control. (**B**) HUVECs treated with conditioned media pictured at different time points not included in Figure 4.

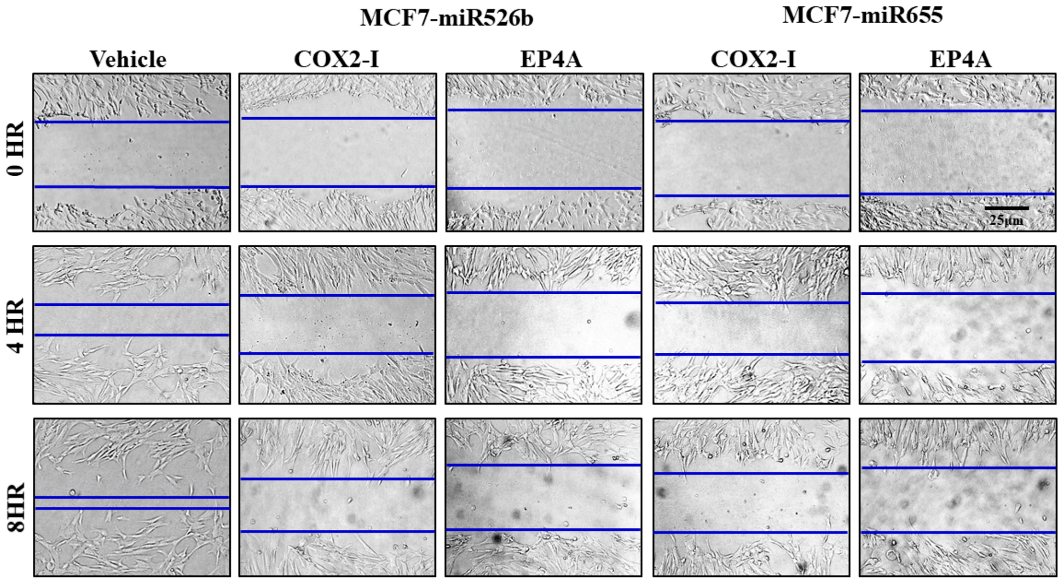


**Figure S5.** HUVEC migration assays. Images from additional time points during treatments with COX2-I or EP4A in addition to MCF7-miR526b or MCF7-miR655 conditioned media.

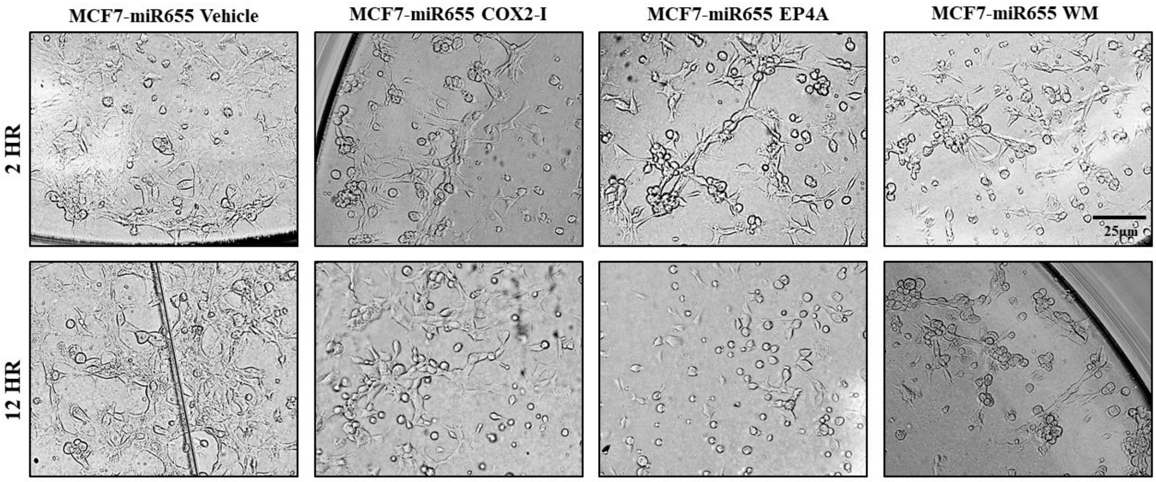


**Figure S6.** HUVEC tube formation assays. Images from additional time points during treatments with COX2-I or EP4A or Wortmannin (WM) in addition to MCF7-miR655 conditioned media.

© 2019 by the authors. Submitted for possible open access publication under the terms and conditions of the Creative Commons Attribution (CC BY) license (http://creativecommons.org/licenses/by/4.0/).
